# Study on the Effect of Ni Addition on the Microstructure and Properties of NiTi Alloy Coating on AISI 316 L Prepared by Laser Cladding

**DOI:** 10.3390/ma14164373

**Published:** 2021-08-04

**Authors:** Yuqiang Feng, Zexu Du, Zhengfei Hu

**Affiliations:** Shanghai Key Laboratory for R&D and Application of Metallic Functional Materials, School of Materials Science and Engineering, Tongji University, Shanghai 201804, China; yuqiang_feng@tongji.edu.cn (Y.F.); 1832875@tongji.edu.cn (Z.D.)

**Keywords:** laser cladding, NiTi alloy, microstructure, wear

## Abstract

This paper investigated 55 NiTi commercial alloy powder and 55 NiTi with 5% pure Ni mixed powder (55 NiTi + 5 Ni) coatings fabricated by laser cladding to study the effect of extra Ni addition on the microstructure and properties of the coating. The XRD and EDS results show that the major phases in the coatings were NiTi and Ni_3_Ti. Besides that, a second phase like Ni_4_Ti_3_, Fe_2_Ti, and NiTi_2_ was also detected, among which, NiTi_2_ was only found in 55 NiTi coating. The proportion of the phase composition in the coating was calculated via the software Image-Pro Plus. The hardness of the cladding layer reaches 770–830 HV, which was almost four times harder than the substrate, and the hardness of 55 NiTi + 5 Ni coating was around 8% higher than that of 55 NiTi coating. The wear resistance of the 55 NiTi + 5 Ni coating was also better; the wear mass loss decreased by about 13% and with a smaller friction coefficient compared with the 55 NiTi coating. These results are attributed to the solid solution strengthening effect caused by Ni addition and the second phase strengthening effect caused by the content increase of the Ni_3_Ti phase in the cladding layer.

## 1. Introduction

NiTi shape memory alloy (55 NiTi or equiatomic NiTi) was discovered by William J. Buehler and his colleagues at the Naval Ordnance Laboratory (NOL) in the late 1950s [1]. 55 NiTi is well known due to its unique superelastic property and shape memory effects (SME) and is widely used in aerospace, automotive, navigation, weapons, and other fields [2,3]. Besides that, the good biocompatibility also makes it outstanding in medical applications [4]. However, the poor workability caused by the high strength, high ductility, and high work hardening of NiTi alloy limits its further extensive utilization [5]. Additive manufacturing (AM), different from conventional processing technologies (e.g., welding and machining) [6,7], which produces components with certain shapes by adding layers of powders progressively [8], provides great potential to expand the application of NiTi alloy.

Laser cladding of NiTi alloy, which produces coating layers on a substrate by melting NiTi powders to improve its surface properties such as hardness, corrosion resistance, and wear resistance [9,10], has become a research hotspot in recent years. B. Norhafzan investigated the surface modification of tool steel by laser cladding with NiTi powder. Results showed that the hardness of the NiTi cladding layer increased three times compared with the substrate [11]. F. Liu prepared NiTi coatings on TA2 substrate (titanium alloy) and found that NiTi, Ni_3_Ti, and NiTi_2_ intermetallic compounds were the main phases in the cladding layer [12]. These mentioned Ni-Ti intermetallics were also observed in the Ni/Ti multilayer film prepared by magnetron sputtering [13,14]. C. Zhang summarized that the B2 phase, tetragonal precipitates, dendrites, and grains are typical microstructures in the NiTi cladding layer due to the rapid heating and cooling cladding process [15]. Furthermore, research on the effect of additives in the NiTi coating is studied. The addition of ZrO_2_ could increase the surface hardness and anti-corrosion performance by laser cladding on AISI 316, as reported by M.L. Lepule [16]. B. Fu studied the effect of Cu addition on the microstructure and properties of NiTi coating, which revealed that the surface hardness increased almost 100% and the wear mass loss in tribological test reduced nearly 50% [17].

Nevertheless, the effect of Ni itself as an additive in the NiTi coating has been neglected. As shown in the Ni-Ti binary phase diagram, 55 NiTi alloy can solve Ni with increasing temperature over 630 °C [18]. If the temperature is further increased up to 1050 °C, a solutionized single phase 60 NiTi with an ordered intermetallic structure is expected. Cooling from this temperature results in precipitation of thermodynamic equilibrium Ni_3_Ti and/or metastable Ni_4_Ti_3_ and Ni_3_Ti_2_ [19]. The 60 NiTi (60 wt.%Ni–40 wt.%Ti) presents higher hardness, excellent corrosion resistance, good biocompatibility, and non-magnetic making it suitable for various applications in aerospace, marine, medical, and food processing industries [20]. Despite the attractive properties mentioned above, 60 NiTi, as a Ni-rich version of the equiatomic NiTi alloy, is also brittle, hard, and difficult to work with [21]. A previous study by K. Khanlari found that 60 NiTi alloy has even better mechanical properties than 55 NiTi [22]. Therefore, it is believed that the performance of 55 NiTi coating could also be further improved with increasing Ni content. In this paper, a mixture powder consisting of 55 NiTi alloy and single Ni powder with a total weight ratio: 60%Ni–40%Ti is used for laser cladding on stainless steel 316 L substrate. The purpose is to study the effect of Ni addition on the microstructure and properties of 55 NiTi coating prepared via laser cladding technology.

## 2. Materials and Methods

### 2.1. Materials Preparation

**Figure 1:**
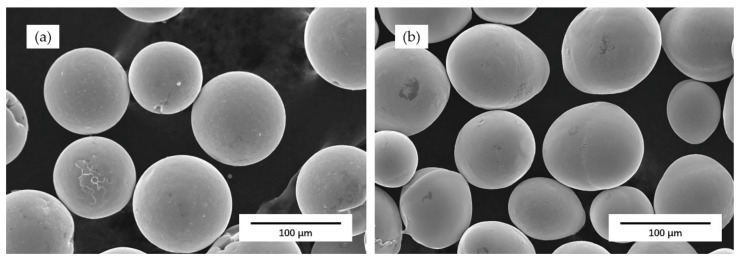
SEM microstructure of powders used in experiment: (**a**) Single Ni powder; (**b**) 55 NiTi alloy powder.

In this work, Ni and 55 NiTi powders (99.9% in purity, Jiangsu Willari New Material Technology Co., Ltd., Xuzhou, China) were selected as raw materials, and their morphologies are shown in Figure 1. The mixed powder was obtained by mixing 55 NiTi alloy powder and pure Ni powder in a weight ratio of 8:1 (equivalent to total weight ratio: 60%Ni–40%Ti) at 250 rpm through a planetary ball mill for 8 h. The chemical composition of these powders is given in Table 1. All powders were dried at 100 °C for 60 min before laser cladding. The 316 L stainless steel with dimension size of 100 mm × 100 mm × 10 mm was used as the substrate, and its chemical composition is given in Table 2. All substrates were polished and ultrasonically cleaned in an acetone bath for 10 min and dried in atmosphere. Prior to laser cladding, substrates were heated up to 250 °C to release the internal stress.

### 2.2. Laser Cladding Process

**Figure 2:**
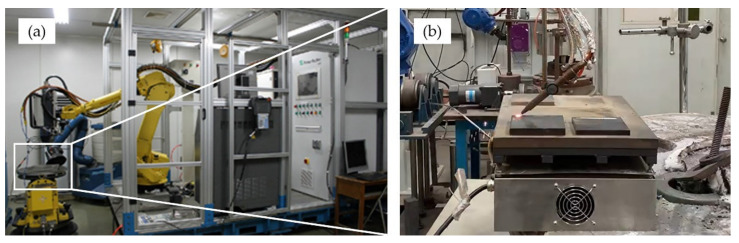
IPG YLS-10000 Laser Equipment: (**a**) Overview of the equipment; (**b**) Working stage.

High-power fiber laser equipment with a beam wavelength of 1070–1080 nm (IPG YLS-10000, IPG Photonics, Oxford, MA, USA) was employed for the laser cladding process (Figure 2). In the experiment, high purity argon with a flow rate of 5 L/min was hired as a shielding gas to prevent the oxidation of the molten metals. In addition, Argon was used as powder feeding gas with 8 L/min flow rate. The diameter of the laser beam was fixed to 0.72 mm. The substrate was placed on the working stage, and powders were added into the feeding system. Laser cladding coatings were fabricated on the substrate under parameters: 2.0 KW power, 2 mm/s laser scanning speed, and 50 rpm powder feeding rate.

### 2.3. Characterization

**Figure 3:**
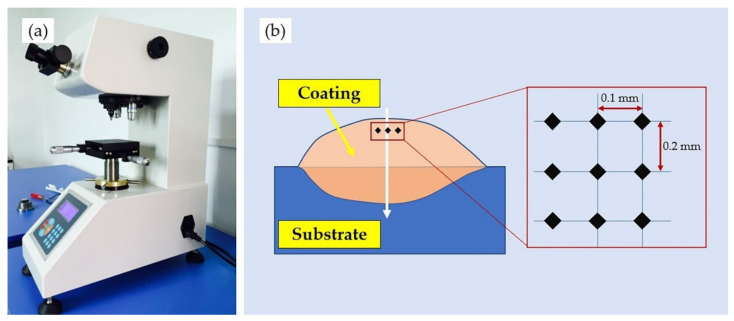
Microhardness tester: (**a**) Overview of the equipment; (**b**) Measurement path.

**Figure 4:**
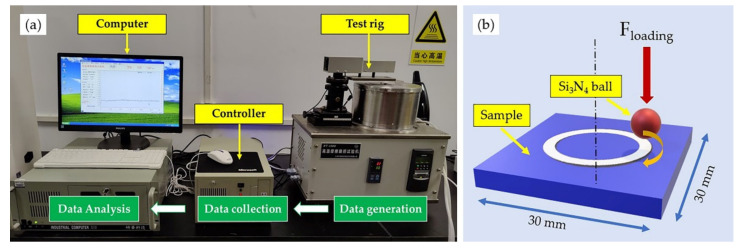
Friction and wear tester: (**a**) Overview of the equipment; (**b**) Schematic illustration of the test rig.

Samples were taken along the direction perpendicular to the laser scanning path. After grinding and polishing, they were etched with an acid liquid (HF:HNO_3_:H_2_O = 1:4:5). An optical microscope observed the microstructure (OLYMPUS GX5, Tokyo, Japan) and scanning electron microscope (SEM, COXEM EM-30, Daejeon, Korea). Meanwhile, an elemental study was conducted using an energy dispersive spectroscopy (EDS, OXFORD Ultim Extreme, Oxford, England). The phase composition was analyzed via X-ray diffraction (XRD, Bruker D8 ADVANCE, Karlsruhe, Germany) at a scanning speed of 10°/min, range from 10° to 80°, with Cu Kα radiation at 40 kV and 40 mA. To research the microhardness development of the cladding layer, as shown in Figure 3, a microhardness tester (HVS-1000A, HuaYin Testing Machine Technology Co., Ltd., Laizhou, China) was employed in the experiment with a load of 1.961 N and holding time of 15 s. Hardness was measured from the coating surface to the substrate at an interval of 0.2 mm and 3 times at the same depth to calculate the average value. The friction and wear properties of the cladding layer were measured using high-temperature friction and wear tester (HT-1000, ZKKH Science and Technology Development Co., Ltd., Lanzhou, China), shown in Figure 4. The experiment was conducted in a ball-on-plate configuration using 5 mm diameter Si_3_N_4_ balls at room temperature with a load of 15 N and a rotation speed of 224 r/min. Samples for wear tests were cut with a size of 30 mm × 30 mm. The system automatically collected the friction coefficient curve while the wear mass loss was measured manually. To obtain more accurate and credible result, each sample was weighed three times.

## 3. Results and Discussion

### 3.1. Macrostructure of the Cladding Layer

**Figure 5:**
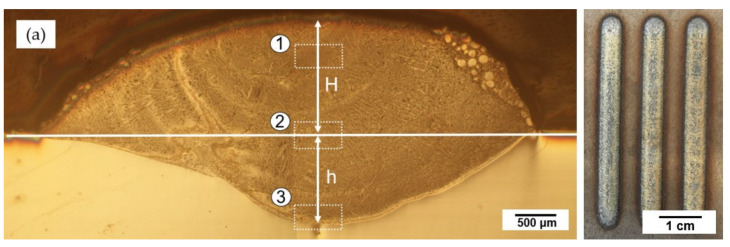

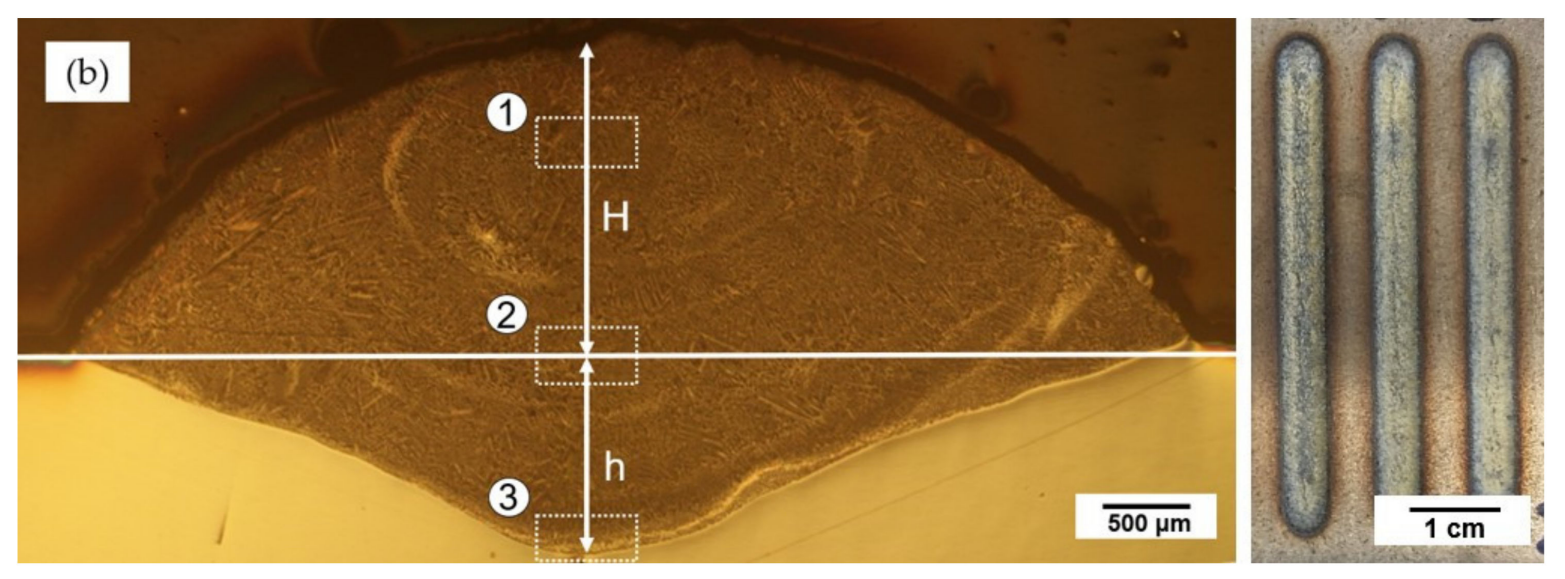
Overview of the laser cladding track and cross-section morphologies of the cladding layer: (**a**) 55 NiTi powder cladding layer; (**b**) 55 NiTi + 5 Ni powder cladding layer.

The laser cladding tracks on the substrate shown in Figure 5 were smooth and clear, without any obvious burr or breakpoint. Looking into the cross-section morphologies, there was no pores or crack found either, and the boundary line between cladding layer and substrate could be easily recognized. However, as shown in Figure 5a, few unfused 55 NiTi powders cladded on the coating surface were found.

Properties of the cladding layer are strongly influenced by the dilution ratio, which the following simplified formula could calculate:

$$\eta =\frac{h}{h+H}$$

where η stands for the dilution ratio, H is the height of the cladding layer, and h is the depth of the molten pool. According to Figure 5, the dilution ratio of the cladding layers is given in Table 3. The dilution ratio of 55 NiTi cladding layer was 0.43, around 13% larger than that of 55 NiTi + 5 Ni layer, which means much more elements (such as Fe and Cr) from substrate SS316 L diffused into the coating.

### 3.2. Microstructure of the Cladding Layer

**Figure 6:**
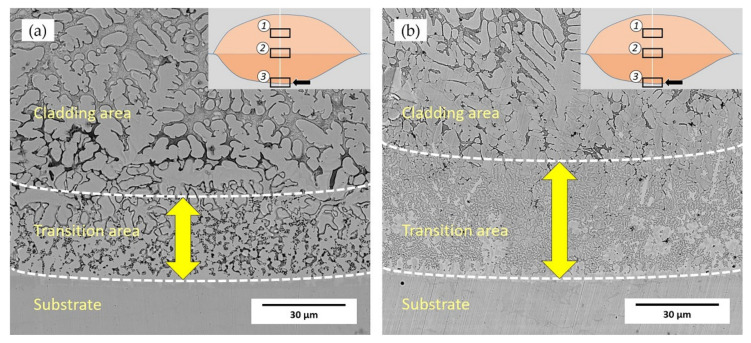
Microstructure of transition area in the cladding layer: (**a**) 55 NiTi powder cladding layer; (**b**) 55 NiTi + 5 Ni powder cladding layer.

The cladding layer is usually divided into three areas: cladding zone, transition zone, and substrate bonding zone [23]. Figure 6 is the SEM micrograph of the transition area, which shows the boundary to the substrate. It can be seen from Figure 6 that the cladding layer has formed good metallurgical bonding with the substrate. The width of the transition zone in the 55 NiTi layer was around 30 μm; however, the width of that in the 55 NiTi + 5 Ni layer exceeded 50 μm.

As they were fabricated under the same parameter (i.e., receiving the same energy per unit square per second), a wider transition zone means more energy went through the cladding area and reached the substrate. Studies reveal that Ni’s content in Ni-Ti alloy could influence its phase transition temperature and properties. The phase transformation temperature of NiTi alloy decreases with the increasing content of Ni [19,24]. That is to say, the energy required for phase transformation reduced, the surplus energy penetrated from the bottom of the molten pool further downwards to substrate, which brought a wider transition area. Due to the rapid heating and cooling of the molten pool, the microstructure formed during the laser cladding process is usually very compact and with intermetallic compounds dissolved in the matrix. The composition undercooling occurs at the frontline of the solidification boundary because of the huge temperature gradient [25]. The formation of microstructure in the cladding layer is mainly affected by temperature gradient G and solidification rate R; thus, G/R is defined as the grain growth factor during the solidification process [26]. In the boundary area to the substrate, the value of G/R is relatively large due to the high-temperature gradient and lower solidification rate. In the meantime, as the substrate is heated to nearly molten status, many particles from the substrate could serve as nucleation sources, which caused a higher nucleation rate than the growth rate in the bottom area. As a result, the solidification process starts from plane grains and gradually changes to columnar grains or coarse dendrites. The coarse dendrites become fine dendrites and equiaxed grains with the solidification frontline moving upwards [27].

**Figure 7:**
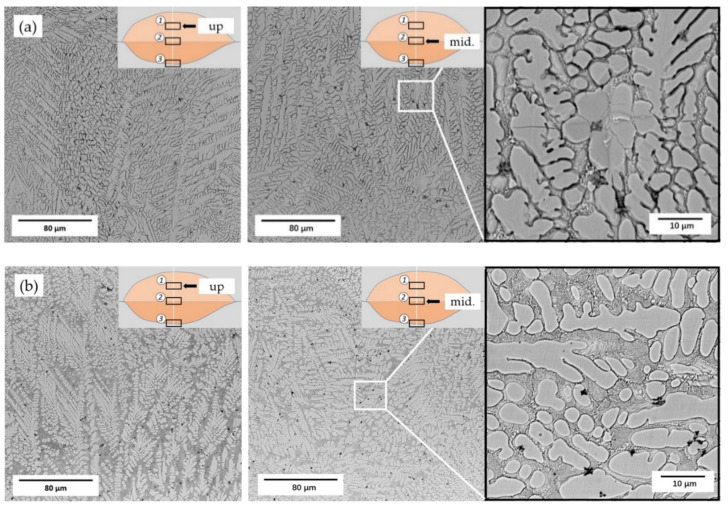
SEM images for upper and middle area microstructure of cladding layer: (**a**) 55 NiTi cladding layer; (**b**) 55 NiTi + 5 Ni cladding layer.

Figure 7 shows the SEM morphologies of the upper and middle area in the cladding layer, in which dendrite is the main structure and its distribution is uniform. The dendritic structures grew at a certain angle and in different directions due to the surface tension at the solidification frontline and the convection effect in the molten pool [28]. The size of the dendritic structure in the upper area is smaller than that in the middle area of the coating. In addition, as shown in Figure 6, the size of the grains in the transition area of 55 NiTi + 5 Ni coating is smaller than that of 55 NiTi, and the microstructure is also denser. It was caused by the addition of extra Ni particles, which could act as embryos as well in addition to NiTi particles, and lead to a higher density of embryos during the nucleation process. The 55 NiTi cladding layer dendrites were more orderly distributed owing to the relative homogeneous nucleation mainly from NiTi particles.

**Figure 8:**
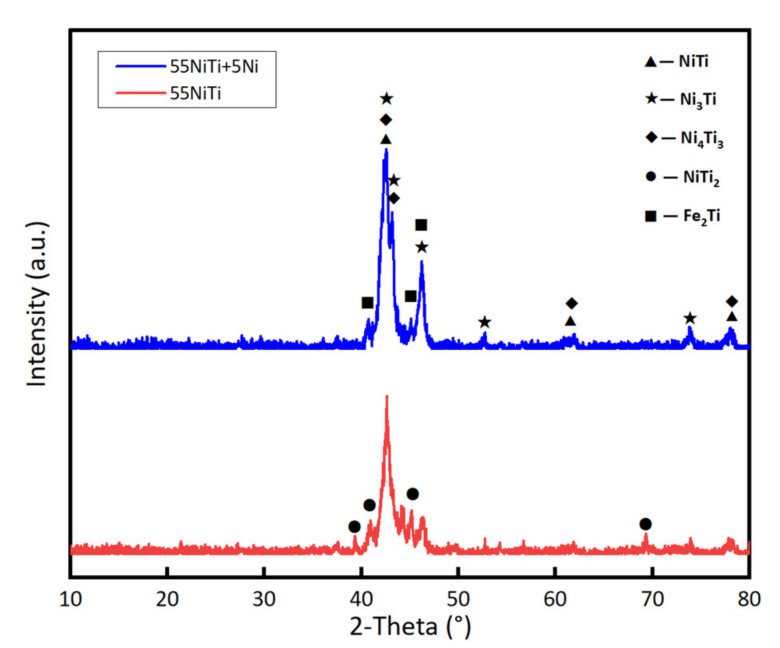
XRD patterns of 55 NiTi and 55 NiTi + 5 Ni laser cladding layer.

Figure 8 shows the XRD pattern of both cladding layers. NiTi and Ni_3_Ti are the major phases in the coating. Besides that, some second phases such as Fe_2_Ti, Ni_4_Ti_3,_ and NiTi_2_ were observed in the cladding layer. Ni_4_Ti_3_ is the metastable phase with rhombohedral crystal structure precipitated from the NiTi phase during the rapid cooling process [19,28], whose diffraction peaks were quite close to NiTi phases. The formation of Fe_2_Ti results from the diffusion of Fe element from the substrate, which was formed during the heating stage based on the Fe-Ni-Ti ternary system at 1000 °C. Due to the high cooling rate at the solidification front line, there was insufficient time for atomic diffusion. Therefore, the Fe_2_Ti phase could be retained at room temperature [29]. The addition of Ni could suppress the formation of the brittle Fe_2_Ti phase [30]. Accordingly, it can be concluded that the Fe_2_Ti phase is mainly distributed in the bottom of the cladding layer.

However, the NiTi_2_ phase was only found in 55 NiTi coating. Due to the rapid heating and cooling process during the laser cladding process, the heat distribution in the molten pool is uneven. The following chemical reactions of Ni-Ti alloy may take place [31]:

Ti + Ni → NiTi

2 Ti + Ni → NiTi_2_

Ti + 3 Ni → Ni_3_Ti

**Figure 9:**
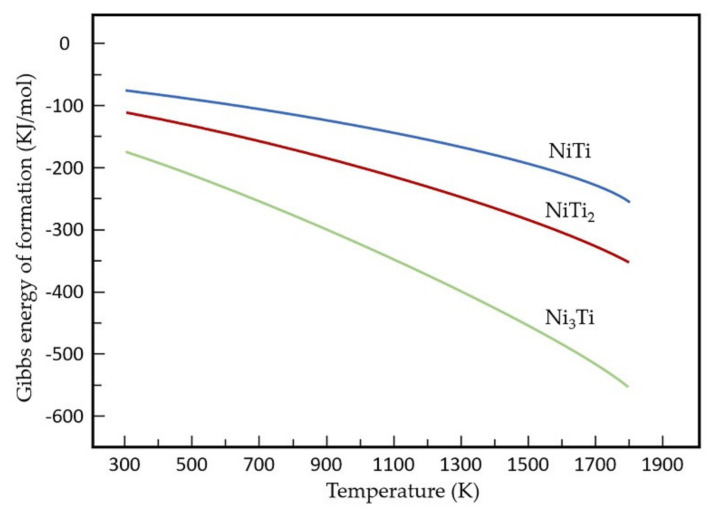
Gibbs free energy of Ni–Ti intermetallic compound.

It is well known that the driving force for the formation of the intermetallic compounds is the difference in their Gibbs free energy. Figure 9 shows the Gibbs energy of NiTi, NiTi_2,_ and Ni_3_Ti for formation at a continuous temperature range. The standard states of the substances are given at room temperature and under normal pressure, and the measurement starts at 300 K with intervals of 100 K. Results are measured and calculated with the software Solgasmix [32,33]. As shown in Figure 9, the driving force for the formation of Ni_3_Ti is stronger than that of NiTi_2_ over a wide temperature range. Therefore, Ni_3_Ti was the first precipitation coming out from the molten pool. It is worth noting that the formation of Ni_3_Ti only occurs by rapid heating and cooling process [14]. During the formation of Ni_3_Ti, each Ti atom took three Ni atoms, the number of Ni atoms decreased quickly. As a result, a Ti-rich region was formed (as shown in Table 4, Region 5), which led to the precipitation of NiTi_2_ subsequently. According to the Ni-Ti binary phase diagram, the formation of NiTi_2_ would take place by peritectic reactions (L + NiTi → NiTi_2_) occurring at around 984 °C and involving a liquid phase [18]. There was no NiTi_2_ phase detected in 55 NiTi + 5 Ni coating, which is closely related to the addition of Ni, which reacts with Ti atom until Ti is depleted. In consequence of the Ni-rich environment in the molten pool, even if a small amount of NiTi_2_ was formed, it would react with surplus Ni atoms and transform to NiTi or Ni_3_Ti phase in the end.

**Figure 10:**
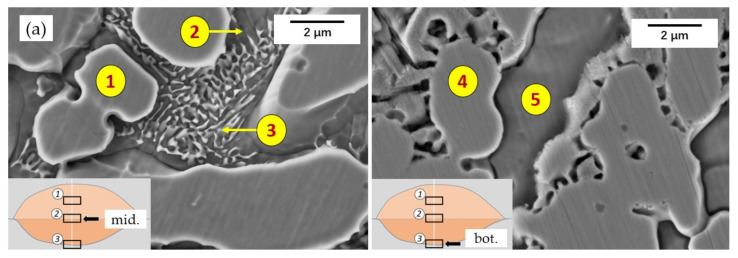

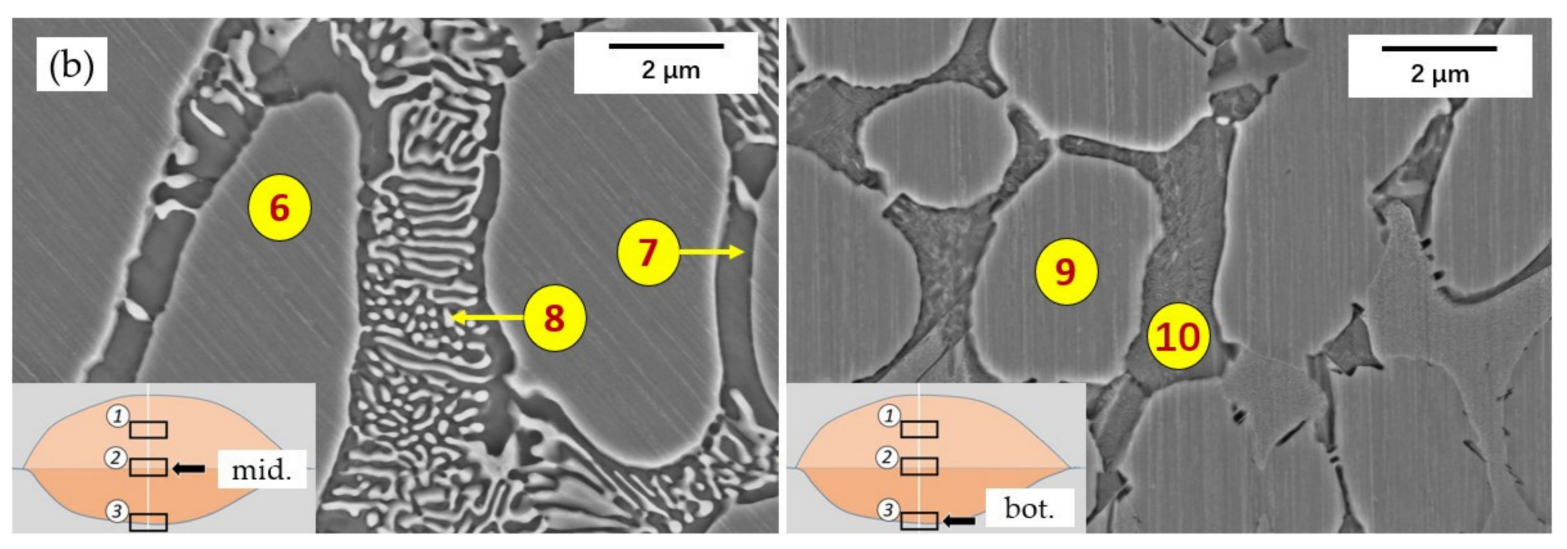
SEM microstructure of middle area and transition area in the cladding layer: (**a**) 55 NiTi cladding layer; (**b**) 55 NiTi + 5 Ni cladding layer.

EDS results of different regions in the coating marked in Figure 10 are listed in Table 4; there are mainly four elements, namely Ni, Ti, Fe, and Cr, in the coating. The massive dendrites in the coating were identified as NiTi phase with B2 structure. At the same time, a certain amount of Fe was also detected in the NiTi phase because the Fe atom has a similar radius and electronegativity to the Ni atom so that the Fe atom could replace some of the Ni atoms in the B2 structure and dissolved in the NiTi phase [34]. Similarly, Cr and Ti have almost the same atom radius and electronegativity, so that the Cr atom could also be more or less dissolved in NiTi phases. The existence of Fe and Cr in the middle area of the coating indicated the sufficient convection and diffusion of the elements in the molten pool during the laser cladding process. According to the XRD results in Figure 8, there might be few metastable Ni_4_Ti_3_ with nanoscale structure precipitated in NiTi phase, but was not observed under SEM, which is supposed to be decomposed to the stable Ni_3_Ti phase through the precipitation sequence of Ni_4_Ti_3_ → Ni_3_Ti_2_ → Ni_3_Ti [35]. According to the EDS result, the dark phase among the dendrites was identified as Ni3Ti (Table 4, Region 2 & Region 7). The white lamellar phase grown on it was a typical eutectic structure of NiTi and Ni_3_Ti. The coexistence of these two phases was observed in the work of Y. Ma during the quick cooling of Ni/Ti multilayer film [14].

**Figure 11:**
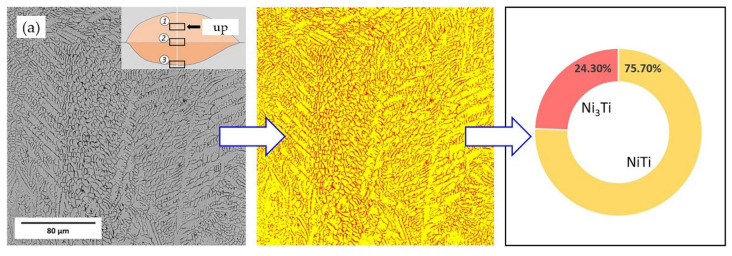

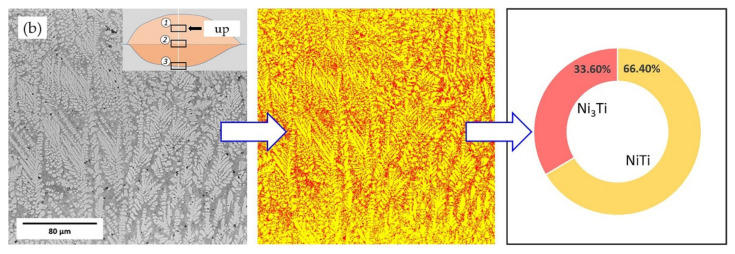
Phase proportion calculation result of the coating upper area via Image-Pro Plus 6.0: (**a**) 55 NiTi cladding layer; (**b**) 55 NiTi + 5 Ni cladding layer.

Thus, it can be seen that NiTi and Ni_3_Ti are the two major phases in the coating, which influence its properties most. The proportion of phase composition between NiTi and Ni_3_Ti was calculated through the software Image-Pro Plus 6.0. Figure 11 is the calculation result of the upper area in the coating. It is clearly visible that 55 NiTi + 5 Ni coating had a higher content of Ni_3_Ti phase with a proportion of 33.60%, which was roughly 10% higher than that of 55 NiTi coating. Consequently, it can be speculated that 55 NiTi + 5 Ni coating has a better surface performance based on the second phase strengthening effect.

### 3.3. Analysis of Properties of Cladding Layer

#### 3.3.1. Microhardness

**Figure 12:**
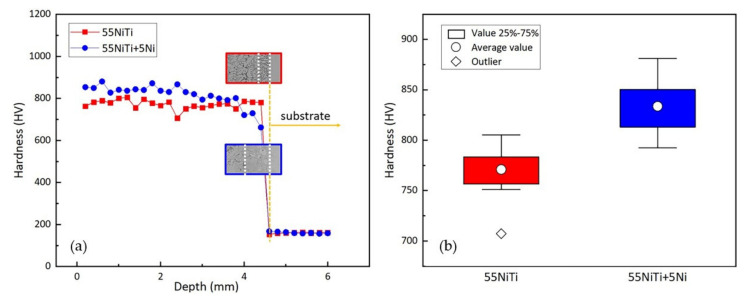
Microhardness results: (**a**) hardness distribution from coating surface to substrate; (**b**) boxplot of the hardness in cladding zone of the coatings.

Figure 12a shows the microhardness distribution of the two cladding layers, which could be clearly divided into three areas: cladding zone, transition zone, and substrate, respectively. The hardness of the cladding layer was significantly improved and reached an average value of 800 HV, which was fast five times harder than the substrate. The main reason for the hardness improvement was that many intermetallic compounds such as Ni_3_Ti and Fe_2_Ti precipitated in the NiTi phase, leading to a second phase strengthening effect. Besides that, the Fe and Cr atom from the substrate are supersaturated in the B2 lattice, which leads to solid solution strengthening.

Comparing the hardness of two coatings, on the one hand, owing to the smaller size and larger density of grains in the cladding layer shown in Figure 7 and higher second phase proportion of Ni_3_Ti, the average hardness of 55 NiTi + 5 Ni coating reached 833 HV, about 8% higher than that of 55 NiTi coating with an average hardness of 771 HV, as shown in Figure 12b. On the other hand, as Table 3 calculated, 55 NiTi coating had a larger dilution ratio, which means more soft phases from substrate diffused into the coating. Beyond that, it was also found in Figure 12a that the hardness of 55 NiTi + 5 Ni coating began to decrease significantly at a depth of 3.8 mm, while that of 55 NiTi coating started to drop at a depth of around 4.4 mm, which is consistent with its wider transition zone observed in Figure 6.

#### 3.3.2. Wear Resistance

**Figure 13:**
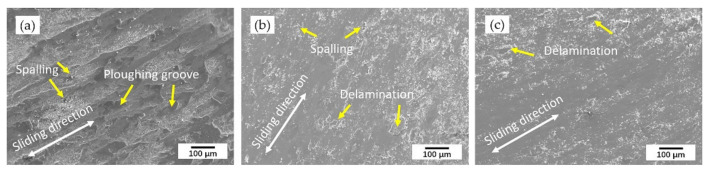
Friction and wear topography of samples: (**a**) SS316 Substrate; (**b**) 55 NiTi coating; (**c**) 55 NiTi + 5 Ni coating.

Figure 13 shows the surface topography of samples after the friction and wear test taken by SEM, in which the wear tracks are very clear to distinguish the sliding directions. Deep and wide plowing grooves oriented along the sliding direction and a small amount of spalling were found on the substrate surface. The deep plowing grooves were the evidence of abrasive wear. While the Si_3_N_4_ grinding ball, which had a hardness of 1300 HV–1500 HV, was around 10 times harder than the substrate. The hard grinding ball under load was readily impressed into the substrate surface and started cutting during the sliding process. Besides that, adhesive wear was also conducted during the grinding ball cutting process. The surface hardness of the coatings improved a lot compared with the substrate due to the fine and dense dendritic structure formed during the rapid cooling process so that the wear track was shallow and narrow, and no plowing groove was found. During the sliding friction, the local fatigue strength of the cladding layer was reduced, and plastic deformation was formed. The mismatch of plastic deformation at different positions led to the delamination and some brittle debonding on the surface. Therefore, delamination wear was dominant in the wear mechanism of the coatings. Furthermore, there was less spalling in the wear surface of 55 NiTi + 5 Ni coating due to its more homogeneous microstructure than 55 NiTi coating.

**Figure 14:**
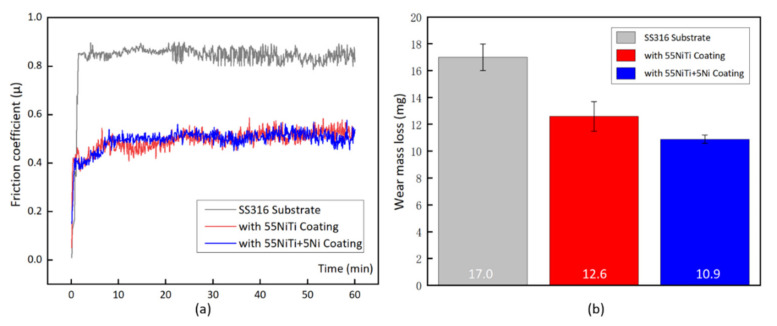
Wear resistance of cladding layers: (**a**) friction coefficient (**b**) wear mass loss.

Table 5 is the result of friction coefficient and wear mass loss from the experiment. Figure 14 is the graphic description of the result, the average friction coefficient of the cladding layer was around 0.5, and the development trend was similar to each other as they shared the same wear mechanism. However, compared to the substrate, the value of friction coefficient decreased over 40%, and wear mass loss reduced about 30% on average, which represented a significant enhancement. As the material surface hardness strongly influences the wear resistance, according to Archard’s Equation, higher surface hardness would lead to better wear resistance [36]. Between the two cladding layers, 55 NiTi + 5 Ni coating displayed a better result in weight loss, about 13% less than 55 NiTi coating, which was in line with the above hardness result. Besides that, the spalling phenomenon found on the wear surface of 55 NiTi coating may attribute to the uneven distribution of the intermetallics in the structure; for instance, as shown in Figure 5a, there are few unfused particles observed in the surface of the 55 NiTi coating.

## 4. Conclusions

The 55 NiTi coating and 55 NiTi + 5 Ni coating were successfully manufactured on the SS316 L substrate via laser cladding technology. The effect of Ni addition on the NiTi coating was studied, and the relationship between its microstructure and surface performance has been discussed. The following conclusions are summarized:

(1) The addition of Ni changed the microstructure and phase composition of the 55 NiTi coating by building a Ni-rich surrounding in the molten pool, which makes all NiTi_2_ phases turn into Ni_3_Ti. As a result, on the one hand, there was no more NiTi_2_ phase found in the 55 NiTi + 5 Ni coating; on the other hand, compared to 55 NiTi coating, the Ni addition increased the proportion of Ni_3_Ti phase by nearly 10% in the 55 NiTi + 5 Ni coating.
(2) The difference between the phase composition of the two coatings has a noticeable impact on the coating surface properties. The addition of Ni raised the surface hardness of 55 NiTi + 5 Ni coating to 830 HV, which was 8% higher than that of 55 NiTi coating and four times harder than the substrate. In addition, the wear resistance has also been improved obviously, the mass loss during friction test reduced 13% compared to 55 NiTi coating.
(3) The enhancement of the coating surface properties is mainly attributed to the increase of second phase proportion and the solid solution strengthening effect caused by the addition of Ni.

## Figures and Tables

**Table 1:** Chemical composition of Ni and 55 NiTi powders (wt.%).

Powders	Ni	Ti	Fe	Nb	Co	C	Si	O
Pure Ni	Bal.	-	0.003	-	0.020	0.020	0.003	0.006
55 NiTi	56.46	Bal.	0.005	0.010	0.005	0.005	-	0.037

**Table 2:** Chemical composition of AISI 316 L stainless steel substrate (wt.%).

Fe	Cr	Ni	Mo	Mn	Si	P	C	S
Bal.	16.32	10.12	2.04	0.92	0.34	0.026	0.016	0.015

**Table 3:** Dilution result of the cladding layers.

Cladding Layer	h/μm	H/μm	Dilution
55 NiTi	1105	1425	0.43
55 NiTi + 5 Ni	1162	1869	0.38

**Table 4:** EDS results of different regions marked in Figure 10.

Coating	Region	Ni (at. %)	Ti (at. %)	Fe (at. %)	Cr (at. %)	Major Phase
55 NiTi	1	26.21	28.59	35.94	9.26	NiTi
	2	56.59	22.74	15.32	5.35	Ni_3_Ti
	3	33.12	27.63	30.66	8.59	Ni_3_Ti + NiTi
	4	17.72	13.80	49.67	18.82	NiTi
	5	17.21	25.08	46.58	11.13	Ni_3_Ti + NiTi_2_
55 NiTi + 5 Ni	6	30.15	28.03	33.69	8.13	NiTi
	7	57.67	24.73	13.86	3.74	Ni_3_Ti
	8	36.28	28.14	27.55	8.03	Ni_3_Ti + NiTi
	9	25.49	25.39	40.14	8.99	NiTi
	10	43.40	18.21	28.62	9.77	Ni_3_Ti

**Table 5:** Result of friction coefficient and wear mass loss.

Sample	Friction Coefficient	Weight Loss (mg)
SS316 Substrate	0.804	17.0
with 55 NiTi Coating	0.514	12.6
with 55 NiTi + 5 Ni Coating	0.501	10.9

## Data Availability

Data sharing is not applicable for this article.
